# Acute Effect on Arterial Stiffness after Performing Resistance Exercise by Using the Valsalva Manoeuvre during Exertion

**DOI:** 10.1155/2015/343916

**Published:** 2015-10-11

**Authors:** Wai Yip Vincent Mak, Wai Keung Christopher Lai

**Affiliations:** Department of Health Technology and Informatics, The Hong Kong Polytechnic University, Hung Hom, Hong Kong

## Abstract

*Background*. Performing resistance exercise could lead to an increase in arterial stiffness. *Objective*. We investigate the acute effect on arterial stiffness by performing Valsalva manoeuvre during resistance exercise. *Materials and Methods*. Eighteen healthy young men were assigned to perform bicep curls by using two breathing techniques (exhalation and Valsalva manoeuvre during muscle contraction) on two separate study days. Carotid pulsed wave velocity (cPWV) was measured as an indicator to reflect the body central arterial stiffness using a high-resolution ultrasound system, and its value was monitored repeatedly at three predefined time intervals: before resistance exercise, immediately after exercise, and 15 minutes after exercise. *Results*. At the 0th minute after resistance exercise was performed using the Valsalva manoeuvre during exertion, a significant increase in cPWV (4.91 m/s ± 0.52) compared with the baseline value (4.67 m/s ± 0.32, *P* = 0.008) was observed, and then it nearly returned to its baseline value at the 15th minute after exercise (4.66 m/s ± 0.44, *P* = 0.010). These findings persisted after adjusting for age, body mass index, and systolic blood pressure. *Conclusion*. Our result suggests short duration of resistance exercise may provoke a transient increase in central arterial stiffness in healthy young men.

## 1. Background

Resistance exercise can be performed using various breathing-control techniques during exertion, and the most commonly adopted breathing-control techniques involve performing exhalation or the Valsalva manoeuvre during exertion. To perform Valsalva manoeuvre, a person must inhale fully and exhale against a closed airway [1]. This process involves the contraction of the diaphragm, abdominal muscles, and other expiratory muscles [2]. As these muscle groups contract, the intra-abdominal pressure increases. Although such an increase in the intra-abdominal pressure is an extravascular event, the pressure is transferred and exerted onto the arterial wall of the abdominal aorta, leading to saturation in the elastin fibre of the abdominal aorta wall and a subsequent increase in the central arterial stiffness [3–5].

Previous studies have demonstrated that performing Valsalva manoeuvre during isometric exercise or heavy weight lifting can alter the hemodynamic function and cause a substantial increase in systolic blood pressure [6, 7]. However, when Valsalva manoeuvre was performed during resistance exercise, systolic blood pressure appeared to be unaffected [8]. Therefore, whether performing resistance exercise could affect central arterial stiffness is still controversial.

## 2. Objectives

The primary objective of this study was to identify the effect of performing Valsalva manoeuvre during resistance exercise on central arterial stiffness. We hypothesized that one bout of resistance exercise in the form of bicep curls combined with Valsalva manoeuvre during exertion can lead to an increase in central arterial stiffness in young men.

## 3. Materials and Methods

### 3.1. Subject

Eighteen healthy young men aged between 20 and 24 years were successfully recruited with informed consent. Subjects who had no history of diabetes, hypertension, cardiovascular disease, chronic kidney disease, or respiratory disease were included in the study. Subjects who took any type of regular medication and smokers were excluded from this study.

### 3.2. Ultrasound Measurement

The local institutional review board approved the experimental protocol. The study included two main experimental sessions. All of the subjects were asked not to consume any food or drink containing caffeine 1 day prior to the study. The two experimental sessions were conducted 1 week apart from each other to eliminate any accumulated effect of postexercise that might affect the outcome of the subsequent experimental session. In each session, all of the subjects were allowed to rest on a bed for 10 minutes before any measurements were taken. The one-repetition maximum (1-RM) test was performed on Day 1 to determine the muscle strength of the subjects based on the maximal weight that the subject could lift. The corresponding weight of the dumbbell (75% of 1-RM) was used in the two experimental sessions [9].

**Figure 1:**
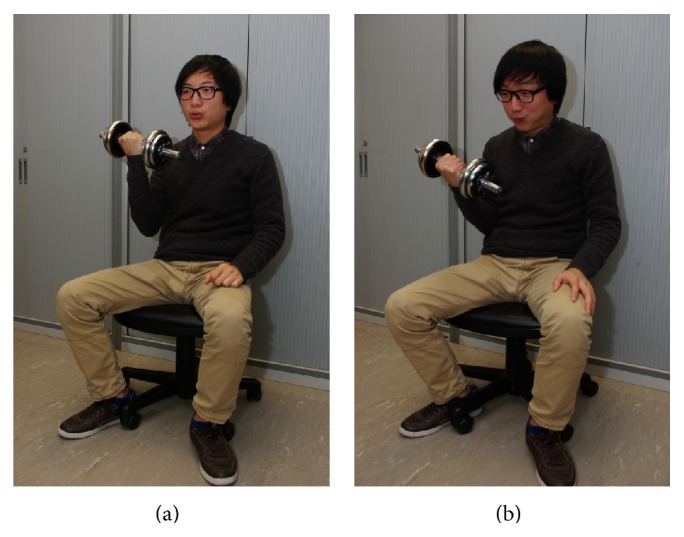
The setup of bicep curls. A volunteer performing bicep curls on Day 1 (a) exhales during exertion and inhales when extending the arm and releasing the dumbbell and on Day 2 (b) performs Valsalva manoeuvre during exertion and exhales when extending the arm and releasing the dumbbell. Strict instruction was provided to every volunteer about the breathing technique during exercise.

In each experimental session, the subjects were instructed to perform ten sets of repeated bicep curls. Each set of repeated bicep curls consisted of completing ten bicep curls within 20 seconds, and a resting period of 90 seconds was given between each set of repeated bicep curls. On Day 1, the subjects were asked to exhale during exertion and inhale when extending the arm and releasing the dumbbell. On Day 2, they were asked to perform Valsalva manoeuvre during exertion and exhale when extending the arm and releasing the dumbbell (Figure 1).

**Figure 2:**
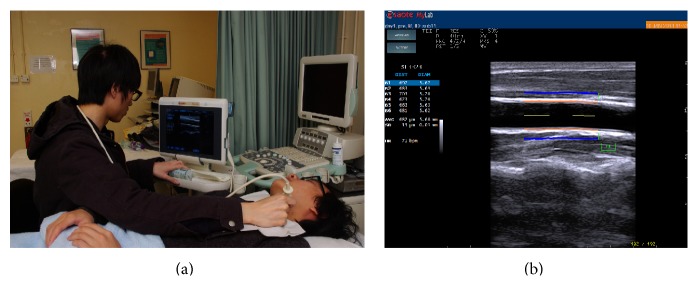
*Ultrasound Scanning of the Carotid Artery*. The setup of the ultrasound measurement of cPWV at a site 1 cm proximal to the bifurcation of the left common carotid artery (a). The measurement interface of RFQAS program (b).

In both sessions, the brachial systolic and diastolic blood pressures were measured using the arm that did not perform the bicep curl. Brachial blood pressure and cPWV were measured at three predefined time intervals: before the exercise (baseline), immediately after the final set of bicep curls was completed (0th min after exercise), and 15 minutes after the final set of bicep curls was completed (15th min after exercise). For obtaining reproducible results, cPWV was always measured at a site 1 cm proximal to the bifurcation of the left common carotid arteries by using the RF-based Quality Arterial Stiffness (RFQAS) software on an Esaote MyLab Sat ultrasound system with a high-frequency linear transducer (Esaote SL3323, 13-6 MHz) (Figure 2). All of the ultrasound examinations were conducted by a single well-trained researcher (VM) to minimize interrater variability.

### 3.3. Data Analysis

All of the collected data were analysed using IBM SPSS (version 21.0.0) software. The average of three cPWV measurements during resistance exercise under different breathing techniques was calculated at three predefined time points (baseline, 0 min after exercise, and 15 min after exercise) and was expressed as mean ± SD and analysed using one-way repeated-measure ANOVA with Fisher's least significant difference post hoc test. The significance level in this study was set at *P* < 0.05.

## 4. Results

The interrater and the intrarater intraclass correlation in the measurement of the cPWV were 0.980 and 0.994, respectively. The subject characteristics are summarized in Table 1. At the 0th minute after resistance exercise was performed using Valsalva manoeuvre during exertion, a significant increase in cPWV (4.91 m/s ± 0.52) compared with the baseline cPWV (4.67 m/s ± 0.32, *P* = 0.008) was observed, and the value then returned to a value near the baseline value at the 15th minute after exercise (4.66 m/s ± 0.44, *P* = 0.010) (Table 2). However, although a small increase in cPWV was observed at the 0th minute after performing resistance exercise by using the exhalation technique during exertion, the rise was shallow and no significant difference in cPWV (*P* = 0.156) among the three time intervals existed (Table 3).

## 5. Discussion

Resistance exercise is a frequently used mode of exercise, particularly among young men, because it enhances muscle strength and body shape. Although patients with coronary heart disease are instructed to avoid performing resistance exercise because it could affect their blood pressure profile and cause arterial stiffness [10, 11], recent studies have proposed that performing aerobic exercises supplemented with resistance exercises could improve the health conditions of stable-condition coronary heart disease patients [10, 12]. Therefore, in addition to being considered as a training plan for healthy people, resistance exercise has also been adopted as a complementary component of rehabilitation programs for patients with cardiovascular diseases [11–13].

Numerous parameters are used to describe arterial stiffness, including pulse wave velocity (PWV), the augmentation index (AIx), distensibility coefficient (DC), and compliance coefficient (CC) [14–16]. Currently, all of these parameters can be easily quantified using the ultrasound imaging method, and PWV is used extensively in scientific research related to cardiovascular function [17]. PWV reflects the speed of the pulsatile blood flow travelling a predefined distance [18]. To quantify central arterial stiffness, the carotid-PWV approach is the most favoured approach because it provides arterial stiffness measurements that can be easily reproduced [15, 19–21]. The result of the present study suggested that performing resistance exercise by using the Valsalva manoeuvre modulated central arterial stiffness, but this change was not significant when resistance exercise was performed using the exhalation technique during exertion.

Therefore, although performing resistance exercise by using Valsalva manoeuvre during exertion is useful and beneficial to experienced weight lifters or athletes when lifting heavy weights with the provision of additional strength and support to the body trunk [2, 8], beginners and amateurs should not use Valsalva manoeuvre in resistance exercises during exertion. Arterial stiffness, one of the markers reflecting the health status of vessel walls [16, 20, 22–24], defines the ability of vessel walls to expand and recoil in response to changes in blood pressure [25]. In a healthy elastic arterial system, high-pressure blood flow can be withstood by the arterial vessel wall through distention during each ventricular contraction [5, 25]. However, when the arteries stiffen, the arterial wall lacks the ability to distend sufficiently. A higher blood pressure must then be generated through stronger contractions of the myocardium to provide an equivalent amount of blood. Ultimately, the long-term practice of resistance exercise by using Valsalva manoeuvre could possibly increase the workload of the myocardium and cause hypertrophy or even promote fibrosis of the myocardium because of the prolonged and frequent abrupt changes in hemodynamics.

## 6. Conclusion

We prospectively investigated the effect of performing resistance exercise on arterial stiffness by monitoring cPWV for 15 minutes after exercise. An acute and significant increase in central arterial stiffness was observed immediately after the subjects performed resistance exercise by using the Valsalva manoeuvre during exertion. However, a similar trend in which central arterial stiffness did not reach a significant level was observed when the subjects performed resistance exercise using the exhalation technique during exertion. Further clinical study is needed to see whether repeated resistance exercise by using Valsalva manoeuvre during exertion could lead to hypertrophy or fibrosis of the myocardium. In addition, since the scope of the present study was limited to including the male subjects and assessing one muscle group, future studies on arterial stiffness in women and other types of resistance exercise for the quadriceps and other muscle groups are warranted.

## Figures and Tables

**Table 1:** The subject characteristics in the study.

Number of subjects	18
Variables	Mean ± SD
Age (yr)	21 ± 1
Weight (kg)	56.0 ± 7.5
Height (cm)	169 ± 6
Brachial systolic pressure (mm Hg)	107 ± 11
Brachial diastolic pressure (mm Hg)	62 ± 8
1-RM (lbs)	20.5 ± 3.3

**Table 2:** Change in cPWV before and after resistance exercise was performed using the Valsalva manoeuvre during exertion.

Time interval	Preexercise	Postexercise	
	Baseline	0th min	15th min
cPWV (m/s)	4.67 ± 0.32^*∗*^	4.91 ± 0.52	4.66 ± 0.44^*∗∗*^

cPWV at three predefined time intervals when subjects performed resistance exercise by using Valsalva manoeuvre during exertion. One-way repeated-measure ANOVA with Fisher's least significant difference post hoc test (with adjustment of age, body mass index, and systolic blood pressure) demonstrated that significant difference in cPWV exists between before exercise and at 0th min (^*∗*^ *P* = 0.008) and between 0th min and 15th min after exercise (^*∗∗*^ *P* = 0.010).

**Table 3:** Change in cPWV before and after resistance exercise was performed using the exhalation technique during exertion.

Time interval	Preexercise	Postexercise	
	Baseline	0th min	15th min
cPWV (m/s)	4.38 ± 0.42	4.64 ± 0.53	4.57 ± 0.55

cPWV at three predefined time intervals when subjects performed resistance exercise by using exhalation technique during exertion. One-way repeated-measure ANOVA (with adjustment of age, body mass index, and systolic blood pressure) demonstrated that no significant difference in cPWV (*P* = 0.156) exists among the three time intervals.
